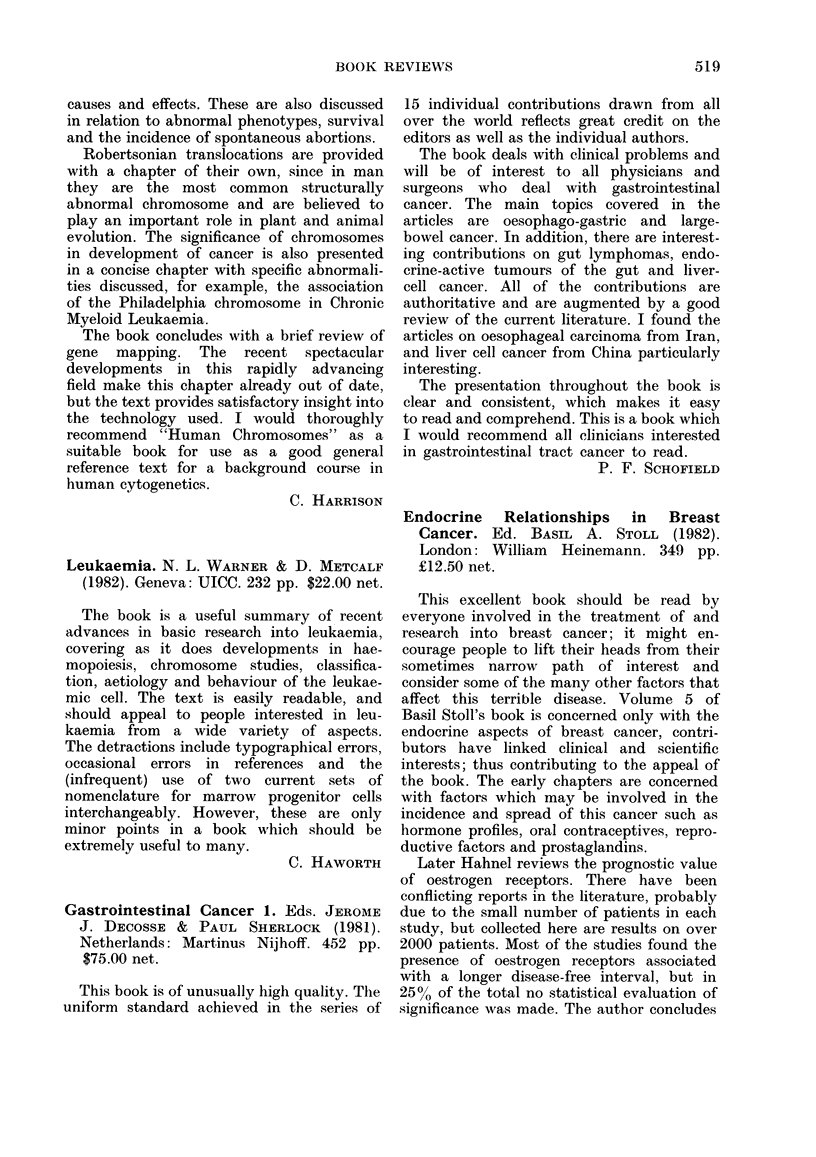# Gastrointestinal Cancer 1

**Published:** 1982-09

**Authors:** P. F. Schofield


**Gastrointestinal Cancer 1.** Eds. JEROME J. DECOSSE & PAUL SHERLOCK (1981). Netherlands: Martinus Nijhoff. 452 pp. $75.00 net.

This book is of unusually high quality. The uniform standard achieved in the series of 15 individual contributions drawn from all over the world reflects great credit on the editors as well as the individual authors.

The book deals with clinical problems and will be of interest to all physicians and surgeons who deal with gastrointestinal cancer. The main topics covered in the articles are oesophago-gastric and large-bowel cancer. In addition, there are interesting contributions on gut lymphomas, endocrine-active tumours of the gut and liver-cell cancer. All of the contributions are authoritative and are augmented by a good review of the current literature. I found the articles on oesophageal carcinoma from Iran, and liver cell cancer from China particularly interesting.

The presentation throughout the book is clear and consistent, which makes it easy to read and comprehend. This is a book which I would recommend all clinicians interested in gastrointestinal tract cancer to read.

P. F. SCHOFIELD